# Adenocarcinoma of the Lung with Miliary Metastases and Primary Resistance Exon 20 Mutations

**DOI:** 10.7759/cureus.3533

**Published:** 2018-10-31

**Authors:** Fred Hsu

**Affiliations:** 1 Radiation Oncology, British Columbia Cancer Agency – Abbotsford Centre, Abbotsford, CAN

**Keywords:** miliary, metastases, lung, exon 20, egfr

## Abstract

Epidermal growth factor receptor (EGFR) exon 20 mutations are seldom tested for in part because they are less common than other EGFR driver mutations and are not associated with sensitivity to EGFR tyrosine kinase inhibitors. We report two cases of lung adenocarcinoma with EGFR exon 20 mutations and a presentation of diffuse, tiny, innumerable lung and brain nodules resembling miliary metastases. Clinicians should be aware of this pattern of presentation in patients with primary resistance EGFR exon 20 mutations.

## Introduction

Exon 19 deletions and exon 21 mutations account for 90% of epidermal growth factor receptor (EGFR) driver mutations and predict for treatment response with EGFR-tyrosine kinase inhibitors (TKI). Exon 20 point mutations comprise only 4% of EGFR mutations and are associated with a reduced sensitivity to EGFR-TKIs [1]. Here we present two patients with primary lung adenocarcinoma and baseline exon 20 primary resistance mutations with diffuse, tiny, innumerable lung and brain nodules resembling a rare pattern of miliary metastases.

## Case presentation

Case 1

**Figure 1:**
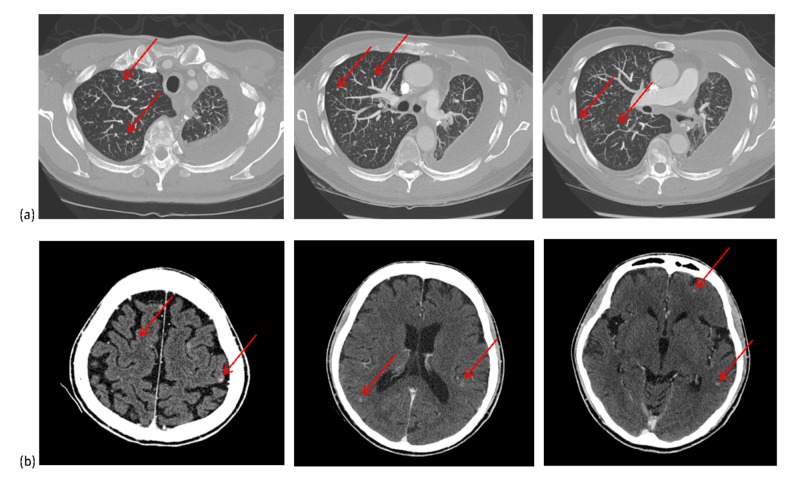
(a) Axial computed tomography (CT) images of the chest and (b) CT images of the brain showing tiny, diffuse, nodular metastases

A never-smoking 64-year-old male of Asian ethnicity presented with stage I disease. He was treated with a left lower lobectomy (stage T1bN0M0). Pathology showed adenocarcinoma histology. Genetic analysis reported no mutation in EGFR exon 19 or 21 using polymerase chain reaction and no anaplastic lymphoma kinase (ALK) re-arrangement using fluorescence in-situ hybridization. He recurred 12 months later with extensive mediastinal lymphadenopathy, a bone metastasis, and a left-sided malignant pleural effusion. Cytology from his pleural fluid showed adenocarcinoma. Genetic analysis showed an EGFR exon 20 variant (c.2313_2314insACG), which results in the insertion of one amino acid residue (p.Asn771_Pro772insThr), and has been associated with reduced sensitivity to EGFR-TKIs [2]. None of the following variants were observed: EGFR exon 19 or 21, v-raf murine sarcoma viral oncogene homolog B (BRAF), kirsten rat sarcoma viral oncogene (KRAS), ALK, isocitrate dehydrogenase (IDH) 1/2, phosphatase and tensin homolog (PTEN), or tumor protein p53 (TP53). The patient was started on cisplatin and pemetrexed followed by maintenance pemetrexed. After 10 months, he developed tiny (2 mm in size), innumerable, diffusely located, bilateral lung nodules on computed tomography (CT) imaging (Figure 1a). There was no predominance in any particular lobe, and there was no dominant pulmonary mass. He received docetaxel (but had an infusion reaction) and then nivolumab for four months with no response and further progression of nodules, confirming that the nodules were disease-related rather than a specific drug-related interstitial disease. He then received single-agent vinorelbine, which had a near-complete effect on the tiny diffuse lung metastases. Another 12 months later, he developed numerous (>15 in number), small (3-6 mm in size), diffusely located intracranial metastases, as identified on a head CT (Figure 1b). There was no significant peritumoral edema and no dominant brain mass. His performance status was deteriorating at that time and he declined whole brain radiotherapy (WBRT). The patient died 25.5 months from the date of metastatic diagnosis from cancer progression.

Case 2

**Figure 2:**
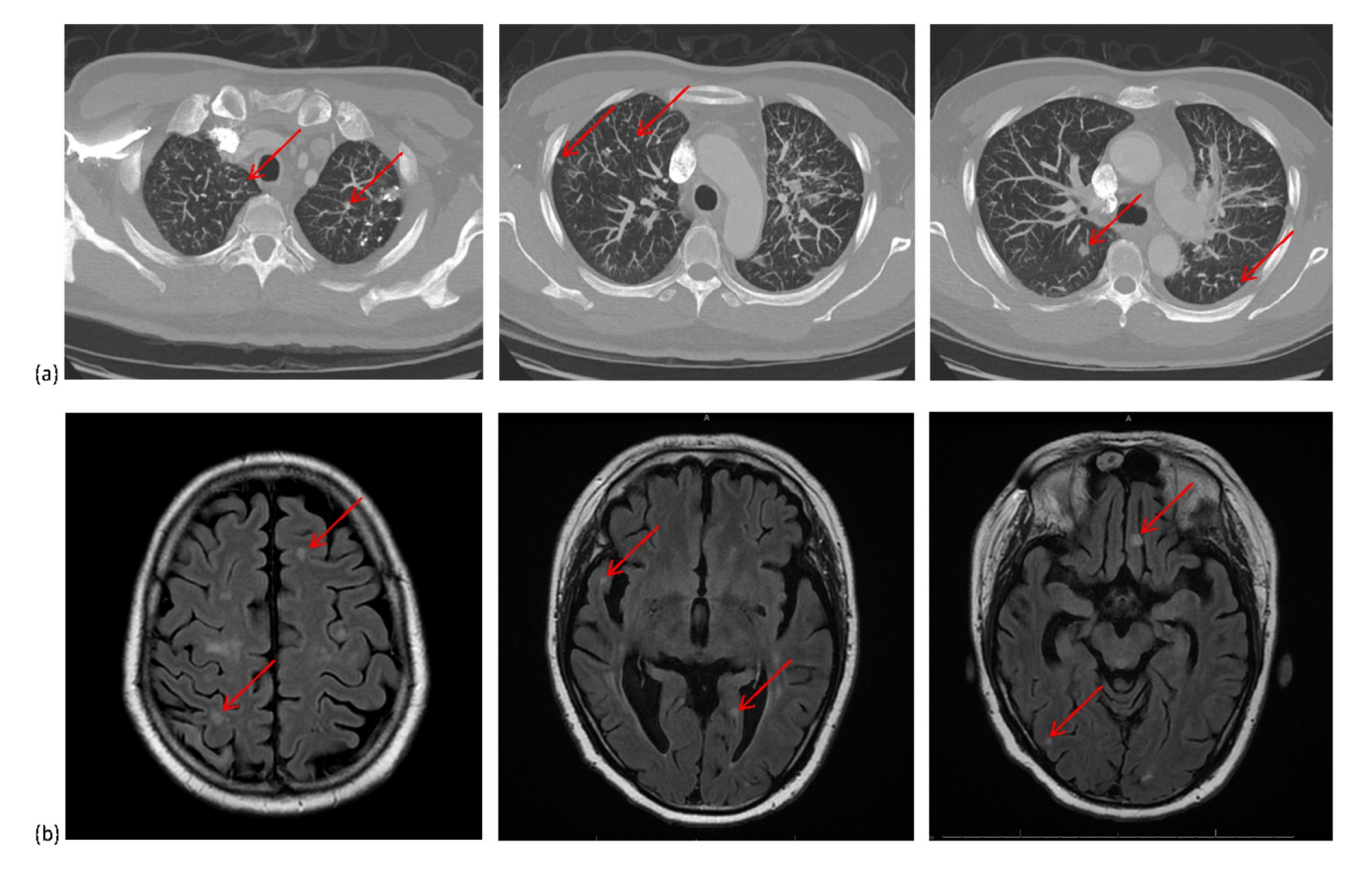
(a) Axial computed tomography (CT) images of the chest and (b) axial magnetic resonance imaging (MRI) of the brain showing tiny, diffuse, nodular metastases

A 47-year-old male of Asian ethnicity and a 17 pack-year history of smoking presented with metastatic disease. Investigations showed a left upper lobe primary tumor (4.7 cm in diameter) with extensive thoracic lymphadenopathy and multiple ring-enhancing brain metastases which measured up to 4.5 cm in diameter predominantly in the right cerebral hemisphere. Pathology obtained from the lung mass showed adenocarcinoma histology. Genetic analysis showed an EGFR exon 20 variant (c.2312_2314dup), which had resulted in the insertion of one amino acid residual (p.Asn771_Pro772insHis), and was associated with reduced sensitivity to EGFR-TKI [2]. None of the following variants were observed: EGFR exon 19 or 21, BRAF, KRAS, ALK, IDH 1/2, PTEN, or TP53. He received WBRT which resulted in a near-complete response in all brain lesions. At the completion of WBRT, re-evaluation with chest CT imaging showed small (8 mm in size), innumerable, diffusely located, bilateral lung nodular metastases (Figure 2a). He received cisplatin and pemetrexed with a partial response of the left lung primary and near-complete resolution of the miliary lung metastases. At progression, he received docetaxel as second-line and nivolumab as third-line and erlotinib as fourth-line therapy. At 15 months, he developed headaches. Magnetic resonance imaging (MRI) of the head showed new tiny (3-8 mm in size), numerous (>25 in number), diffusely located, nodular brain metastases with no significant peritumoral edema or dominant mass lesion (Figure 2b). He was treated with repeat WBRT. The patient died 24 months from the time of initial diagnosis from cancer progression.

## Discussion

We present two cases of tiny, innumerable, diffuse lung and brain metastases in patients with lung cancer and exon 20 mutations. This presentation of miliary metastases is uncommon [3]. In both cases, the mutation was present at baseline (germinal mutation or somatically mutated subclone) rather than acquired secondary to EGFR-TKI resistance. To our knowledge, this is the first case report of miliary brain and miliary lung metastases seen together in EGFR exon 20 mutant lung adenocarcinoma. 

Driver mutations in exon 19 and 21 comprise the majority of EGFR mutations and are more frequently tested for because of their therapeutic significance. Laack et al. described a miliary pattern of pulmonary metastases in five patients with lung adenocarcinoma and exon 19 deletions [4]. A similar pattern of miliary metastases in the brain in patients with exon 19 deletions was reported by Sekine et al [5]. Wu et al. examined patients presenting with miliary pulmonary carcinomatosis and found higher rates of adenocarcinoma with EGFR (exon 18-21) mutations, and suggested that EGFR-TKI may be the treatment of choice for non-small cell carcinoma patients with a miliary pattern of disease [6]. Finally, a recent population-based study showed an increased incidence of miliary lung and brain metastases in patients with lung cancer carrying an EGFR exon 19 or 21 mutation [7].

Lung adenocarcinoma with exon 20 mutations may predispose to the same miliary pattern but are not associated with sensitivity to EGFR-TKI. Our literature search found two cases of distinct exon 20 point mutations and the same pattern of presentation. Schaller et al. reported a case of miliary lung metastases in a patient with a baseline T790M (exon 20) mutation [8]. Ruppert et al. reported a case of miliary brain metastases in a patient with a different exon 20 deletion (c.2297_2305dupTGGCCAGCG) [3]. Interestingly, Falk et al. reported a case of miliary brain and pulmonary metastases in a patient with lung adenocarcinoma and an echinoderm microtubule-associated protein-like 4 (EML4)-ALK translocation [9].

Miliary metastases could be a pattern of metastatic behavior associated with more than a few driver mutations. Since not all EGFR mutations confer sensitivity to EGFR-TKI therapy, not all patients with a miliary presentation may respond to EGFR-TKIs. In patients with miliary metastases and the absence of an EGFR exon 19 or 21 mutation, analysis of less common driver mutations should be considered. An underlying driver mutation may be present which may have therapeutic significance. For instance, patients with an EGFR exon 20 T790M mutation may have a better response with osimertinib over first- or second-generation EGFR-TKIs [10].

## Conclusions

There is growing literature associating distinct driver mutations with miliary metastases. Clinicians should beware of this pattern of presentation in patients with EGFR exon 20 mutations because they are resistant to EGFR-TKIs.
